# Assessment of German Public Attitudes Toward Health Communications With Varying Degrees of Scientific Uncertainty Regarding COVID-19

**DOI:** 10.1001/jamanetworkopen.2020.32335

**Published:** 2020-12-10

**Authors:** Odette Wegwarth, Gert G. Wagner, Claudia Spies, Ralph Hertwig

**Affiliations:** 1 Center for Adaptive Rationality, Max Planck Institute for Human Development, Berlin, Germany; 2 Institute of Medical Sociology and Rehabilitation Science, Charitè–Universitätsmedizin Berlin, Berlin, Germany; 3 Socio-Economic Panel Study (SOEP), Berlin, Germany; 4 Center for Anesthesiology and Operative Intensive Medicine, Charitè–Universitätsmedizin Berlin, Berlin, Germany

## Abstract

This survey study assesses attitudes of the German public regarding COVID-19 health communications with varying degrees of scientific uncertainty.

## Introduction

The coronavirus disease 2019 (COVID-19) pandemic has exposed scientific uncertainty in its raw form. When facts are uncertain, policy makers and health experts sometimes shy away from communicating scientific uncertainty,^1^ fearing that the uncertainty will generate mistrust.^2^ In Germany, for instance, the pandemic-related threat scenarios invoked have sometimes been devoid of uncertainty.^3^ Nevertheless, presenting uncertain aspects of the pandemic as certain may adversely affect citizens’ trust and compliance with containment measures should those reports later prove invalid.^4^ We assessed people’s preferences for health communications with varying degrees of scientific uncertainty in the context of the COVID-19 pandemic and explore factors associated with the preferred form of communication.

## Methods

A sample group of German residents 18 years or older were surveyed from July 13 through July 20, 2020, to investigate their preferences for communications regarding COVID-19 (Table). Of the 3182 people invited to take the survey, 744 did not respond, 47 did not complete the survey questionnaire, 380 were excluded after failing a quality check (eg, people who completed the survey too quickly), and 2011 completed the survey, yielding a response rate of 71.8% (2011 of 2802 [after deleting the 380 excluded questionnaires from the original 3182]). The sample was randomly drawn from the PAYBACK Online Panel (Munich, Germany) by the market research institute Infratest dimap (Berlin, Germany). The panel consists of more than 80 000 panelists who are continuously recruited by off-line invitation only (to reduce self-selection bias) from the 25 million members of the PAYBACK Germany loyalty scheme. Owing to the high standard of panel recruitment and the structure of the panelists, Infratest dimap uses the panel for social science studies. The ethics committee of the Max Planck Institute for Human Development approved the content and design of the study. Written informed consent, granted by waiver by the ethics committee of the Max Planck Institute for Human Development, was obtained online from all participants before the study. This study followed the disclosure requirements of the Code of Professional Ethics and Practice of the American Association for Public Opinion Research (AAPOR) reporting guideline (eMethods 3 in the Supplement).

**Table.** Demographic Characteristics of the Survey Sample Compared With the 2018 Microcensus of the German Federal Statistical Office

Characteristic	Survey sample, No. (%) (N = 2011)^a^	Microcensus 2018, %^a^
Females	1014 (50.4)	51.4
Age, y		
18-29	225 (11.2)	15.2
30-39	283 (14.1)	14.1
40-49	300 (14.9)	14.2
50-59	438 (21.8)	20.2
≥60	765 (38.0)	36.3
Educational level		
Lower secondary education	292 (14.5)	31.5
Middle school	975 (48.4)	32.2
High school	740 (36.8)	33.3
No qualifications	6 (0.3)	4.1
Region		
East	413 (20.5)	20.8
West	1598 (79.5)	79.2

^a^ Percentages are rounded and may not total 100.

Participants were shown 4 scenarios in random order that communicated information regarding the COVID-19 pandemic (eg, deaths, reproduction numbers) with varying magnitudes of scientific uncertainty (eMethods 2 in the Supplement). All numbers used in the scenarios were based on the actual numbers of people with positive test results for severe acute respiratory coronavirus 2, COVID-19 related deaths, and the reproduction number in Germany, drawn from the daily reports of the Robert Koch Institute (Berlin, Germany) from March 22 to April 2, 2020. Categorization of the magnitude of uncertainty was based on a systematic review^1^ (eMethods 1 and the eFigure in the Supplement). Participants ranked the scenarios according to (1) which form of communication they would most prefer government and health experts to use regarding the COVID-19 pandemic and (2) their potential to motivate support and compliance with containment measures such as social distancing. The participants’ basic numeracy was measured by the scale from Schwartz et al^5^ and was judged to be present if all 3 questions were answered correctly.

Statistical analyses were performed with SPSS Statistics 26 software (IBM). Data were weighted by age, sex, region, household size, and educational level according to the 2018 Microcensus of the German Federal Statistical Office to approximate the representativeness of the target population. Summary statistics are provided as proportions with 95% CIs. The χ^2^ test was used to compare differences in informational preferences within groups, and the Mann-Whitney test was used to test for differences between groups. Multinomial logistic regression was used to investigate the associations between the preferred form of COVID-19 communication and demographic characteristics. *P* values were 2-sided, with statistical significance set at *P* < .05 for single comparisons and at *P* < .015 for multiple comparisons, after Bonferroni correction.

## Results

The mean age of the 2011 participants was 52.0 years (range, 18-89 years); 1014 (50.4%) of the respondents were women. The Table presents additional demographic characteristics.

Of the 2011 respondents, significantly more (650 respondents [32.3%; 95% CI, 30.3%-34.4%]) chose the scenario expressing the highest magnitude of uncertainty (verbal and numerical uncertainty) as their preferred form of communication regarding the COVID-19 pandemic (Figure) than any of the other scenarios. We compared scenario 1 (highest uncertainty) with each of the other 3 (scenario 1 vs 2, χ^2^_1_ = 265.094, *P* < .001; scenario 1 vs 3, χ^2^_1_ = 256.823, *P* < .001; scenario 1 vs 4, χ^2^_1_ = 318.947; *P* < .001). Approximately one-fourth of the respondents (501 [24.9%; 95% CI, 23.0%-26.8%]) chose the scenario explicitly denying uncertainty, 435 (21.6%; 95% CI, 19.8%-23.4%) chose the scenario expressing verbal uncertainty only, and 425 (21.1%; 95% CI, 19.3%-22.9%) chose the scenario that left uncertainty unmentioned.

**Figure.**
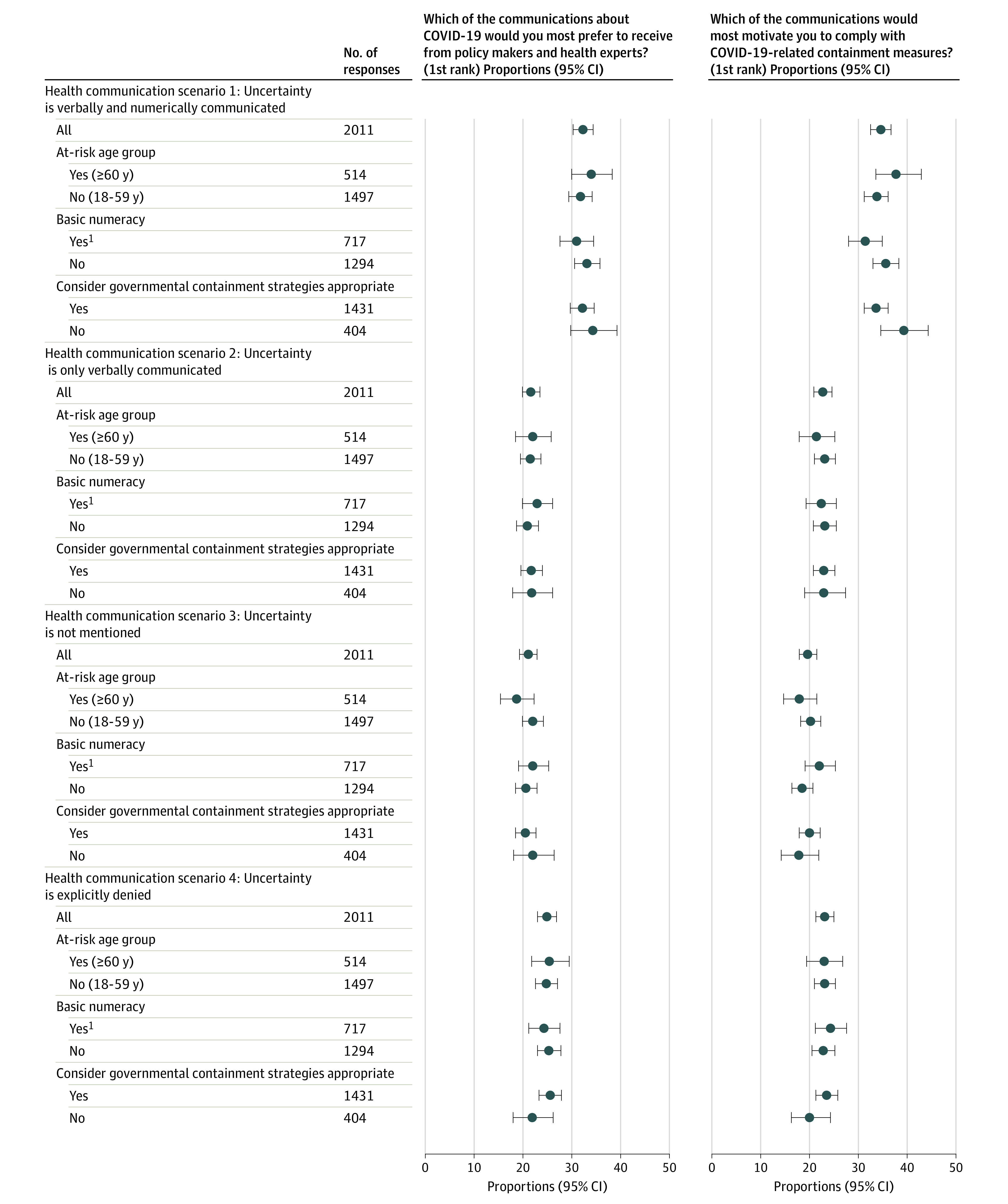
Participants’ First-Ranked Choices for the Preferred Magnitude of Scientific Uncertainty in Communication Regarding the Course of the COVID-19 Pandemic and for Motivating Compliance With Containment Measures Percentages are rounded and may not total 100. The respondents’ basic numeracy was measured by the scale from Schwartz et al^5^ and was judged to be present if all 3 questions were answered correctly. The whiskers indicate 95% CIs.

A similar ranking—with an even more pronounced preference for the scenario expressing the highest magnitude of uncertainty—was observed for the form of communication participants judged most likely to motivate them to support and comply with pandemic containment measures (Figure). Participants who deemed current governmental COVID-19 containment strategies to be exaggerated (n = 404) indicated they would be more inclined to comply with containment measures when presented with communication expressing the highest magnitude of uncertainty than were those who considered these strategies appropriate (n = 1431) (39.4%; 95% CI, 34.6-44.3 vs 33.6%; 95% CI, 31.2-36.0) (difference, 5.8%; 95% CI, 4.1-7.5; *P* = .015).

Basic numeracy,^5^ belonging to an at-risk age group (≥60 years), sex, and educational level were not associated with the preferred form of COVID-19 communication.

## Discussion

In this survey of German residents, a majority of respondents indicated a preference for open communication of scientific uncertainty in the context of the COVID-19 pandemic. For those who are currently skeptical of governmental containment measures, communication expressing uncertainty appeared to be particularly effective in motivating them to comply with the measures. The generalizability of these results may be limited by our sample, which consisted of only German residents. These results are unexpected to the extent that research in other nonmedical and medical domains suggests that the communication of uncertainty prompts avoidance and increased levels of discomfort.^2,6^ We speculate that our respondents—and perhaps people worldwide—may be more open to the communication of uncertainty in the context of COVID-19 because the individual and collective experience of the pandemic is one of rapidly changing knowledge and absence of certainty. It may even be that admitting and communicating scientific uncertainty to the public fosters trust.

## Notes

Supplement. **eMethods 1.** Background Information on Survey Construction **eFigure.** Expressions for Communicating the Magnitude of Scientific Uncertainty, in Order of Decreasing Precision **eMethods 2**. Survey Questionnaire **eMethods 3.** AAPOR Survey Disclosure Checklist
