# Supplementary material for: Assessment of German Public Attitudes Toward Health Communications With Varying Degrees of Scientific Uncertainty Regarding COVID-19
Source: JAMA Netw Open. 2020 Dec 10;3(12):e2032335. doi: 10.1001/jamanetworkopen.2020.32335 (PMC7729432; doi:10.1001/jamanetworkopen.2020.32335)
Supplement: Supplement. — eMethods 1. Background Information on Survey Construction eFigure. Expressions for Communicating the Magnitude of Scientific Uncertainty, in Order of Decreasing Precision eMethods 2. Survey Questionnaire eMethods 3. AAPOR Survey Disclosure Checklist [file jamanetwopen-e2032335-s001.pdf]

SUPPLEMENTARY ONLINE CONTENT

Wegwarth O, Wagner GG, Spies C, Hertwig R. Assessment of German public attitudes toward health communications with varying degrees of scientific uncertainty regarding COVID-19. *JAMA Netw Open.* 2020;3(12):e2032335. doi.10.1001/jamanetworkopen.2020.32335

eMethods 1. Background Information on Survey Construction

eFigure. Expressions for Communicating the Magnitude of Scientific Uncertainty, in Order of Decreasing Precision

eMethods 2. Survey Questionnaire

eMethods 3. AAPOR Survey Disclosure Checklist

This supplementary material has been provided by the authors to give readers additional information about their work.

eMethods 1. Background information on survey construction (categorization of the magnitude of uncertainty in communication; numbers used in the scenarios)

Categorization of the magnitude of uncertainty in communication

Our categorization of the magnitude of uncertainty expressed in the four scenarios was based on a systematic review by van der Bles et al., which proposed a comprehensive framework for communicating scientific/epistemic uncertainty. According to this framework, expressions of scientific uncertainty can range from giving a full explicit probability distribution as the highest level to explicit denial that uncertainty exists as the lowest level (**Figure 1**). We decided against including level (i) because these forms of expressing uncertainty (a full explicit probability distribution or summary of the probability distribution) may be understood in scientific communication but not necessarily by laypeople, the targets of our survey. We therefore began by presenting one candidate scenario at level (ii), which included numbers (eg, ranges) and a verbal statement of uncertainty (eg, "it cannot be said for certain"), and one scenario at level (iv), which included a quantifying verbal statement (eg, "probably"). Two further candidate scenarios expressed a higher degree of scientific certainty, one at level (vii), leaving scientific uncertainty unmentioned, and the other at level (viii), explicitly denying uncertainty by using, for instance, qualifying verbal statements such as "no doubt."

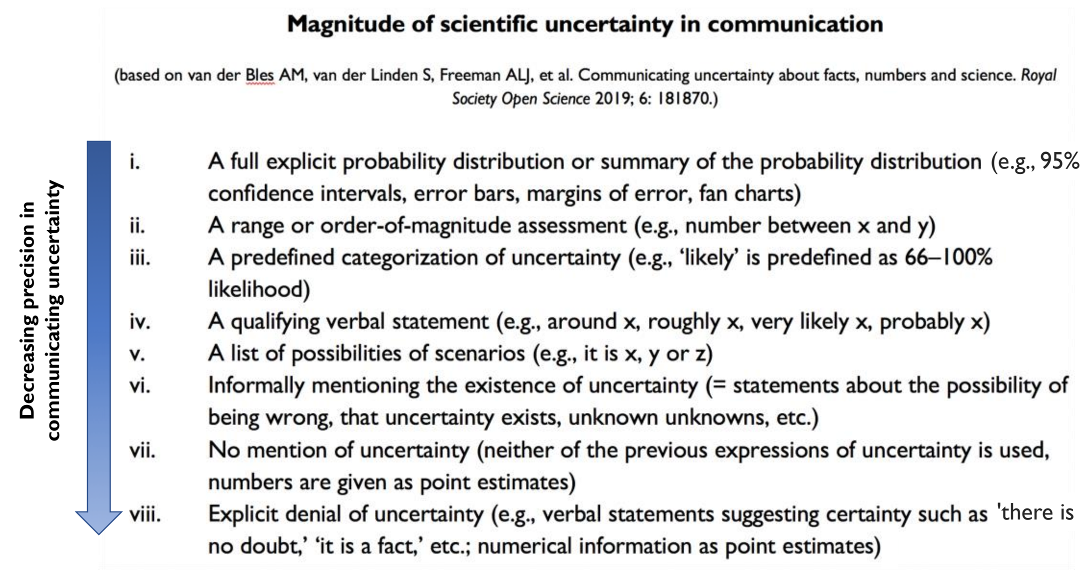

eFigure. *Expressions for communicating the magnitude of scientific uncertainty, in order of decreasing precision (capability of expressing detail of magnitude)*

Given that communication about the COVID-19 pandemic in Germany commonly involves a presentation of data that are set in context by health experts, each scenario presents the scientific

© 2020 Wegwarth et al. *JAMA Network Open.*

facts, followed by an expert commentary. This commentary always reflects the intention of what the "facts part" was meant to communicate. For instance, in the scenario depicting the facts as being uncertain, the subsequent expert commentary confirms this depiction.

To the best of our knowledge, our survey is the first to systematically investigate the effect of communicating information with varying magnitudes of scientific uncertainty.

Numbers used in the scenarios

All numbers used in the scenarios were based on the actual numbers of people testing positive for SARS-CoV-2, COVID-19 related deaths, and the reproduction number in Germany, drawn from the daily reports of the Robert Koch Institute (Belin, Germany) from March 22 to April 2, 2020.

© 2020 Wegwarth et al. *JAMA Network Open.*

eMethods 2. Survey questionnaire

[Start survey]

Let us now come to an important question that is occupying policy makers and health experts as well as many citizens and is being debated intensively: How should the available information on the course of the coronavirus pandemic be communicated to the public so that it is best understood and so that measures for containing the pandemic (e.g., wearing a face mask) are best supported? To answer this question, we need your help. We will now show you four texts that present the possible future course of the coronavirus pandemic in different ways. First, please read each text carefully. We will then ask you some questions.

[Presentation of scenarios in random order]

***[Scenario 1 | Highest magnitude of uncertainty**: Uncertainty is expressed by probability distributions (e.g., ranges, confidence interval) and qualifying verbal statements (e.g., unlikely, is not certain, probably, etc.)]*

In the past few days, the number of infections in Germany has increased again, from 1,018 to 2,337 positive tests per day last week to 2,082 to 3,609 positive tests per day this week. This means that the reproduction number (R), which was between 0.7 and 0.9 last week, is now between 0.8 and 1.3. There was a similar trend in the number of COVID-19 related deaths. Whereas between 110 and 227 infected people died each day last week, between 126 and 315 people died each day this week.

Experts emphasize that these data do not necessarily indicate an upward trend, as there is some overlap between the case numbers, which fluctuate daily. It is therefore uncertain whether the differences observed between the two weeks are due to random fluctuation or are the first warning signs of the onset of a second wave of coronavirus infections. However, because a second wave cannot be ruled out either, experts recommend the continued use of protective measures such as wearing face masks in public places in order to protect risk groups.

***[Scenario 2 | Intermediate magnitude of uncertainty**: Uncertainty is expressed by qualifying verbal statements (e.g., unlikely, is not certain, probably etc.)]*

In the past few days, the number of infections in Germany has probably increased again. There may also be a similar trend in the number of COVID-19 related deaths and in the reproduction number. The reproduction number indicates the number of people infected by a single person with COVID-19.

Experts emphasize that these data do not necessarily indicate an upward trend, as there is some overlap between the case numbers, which fluctuate daily. It is therefore uncertain whether the

© 2020 Wegwarth et al. *JAMA Network Open.*

differences observed between the two weeks are due to random fluctuation or are the first warning signs of the onset of a second wave of coronavirus infections. However, because a second wave cannot be ruled out either, experts recommend the continued use of protective measures such as wearing face masks in public places in order to protect risk groups.

***[Scenario 3 | No mention of uncertainty**: Neither numbers (which are usually given as point estimates) nor verbal statements suggest uncertainty]*

In the past few days, the number of infections in Germany has risen again, from 1,582 positive tests per day last week to 2,827 positive tests per day this week. This means that the reproduction number (R), which was 0.8 last week, is now back at 1.1. There was a similar trend in the number of COVID-19 related deaths. Whereas an average of 170 infected people died each day last week, the daily death rate for this week is already at 224 people.

Experts emphasize that this increase is cause for concern and indicates the beginning of a second wave of infections. For that reason, they recommend the continued use of preventive measures such as wearing a face mask in public places in order to protect risk groups.

***[Scenario 4 | Explicit denial of uncertainty**: Use of qualifying verbal statements suggesting certainty (e.g., there is no doubt, it is clear, it is a fact etc.) and of extreme numbers (upper bounds of distributions)]*

In the past few days, the number of infections in Germany has risen again. Whereas there were up to 2,337 positive tests per day last week, this week there were up to 3,609 positive tests per day. The reproduction number has also risen again to 1.3, which means that each person infected with the coronavirus infects more than one other person. There was a similar trend in the number of COVID-19 related deaths, which rose to as many as 315 per day.

Experts emphasize that this development in case numbers leaves no doubt that a second wave of infections has already begun. For this reason, continued use of preventive measures such as wearing a face mask in public places in order to protect risk groups is of paramount importance.

[Question for outcome 1 | *Preferred form of communication about aspects of the COVID-19 pandemic*]

In your opinion, which of form of communication by policy makers and health experts is most suitable for informing people about course of the COVID-19 pandemic? Please rank the four alternatives in order of your preference from 1 (= "most preferred") to 4 (= "least preferred").

© 2020 Wegwarth et al. *JAMA Network Open.*

[Question for outcome 2 | *Compliance with preventive measures*]

Now please think back on the containment measures you have experienced in the past weeks, such as restrictions on social contacts, shutdowns of schools and daycare facilities, social distancing, and wearing face masks. If there were a second wave of coronavirus infections, which of the four texts would most likely motivate you to support and comply with these containment measures, and which would least motivate you? Please rank the four alternatives from 1 (= "Would most motivate me to comply with the measures") to 4 (= "Would least motivate me to comply with the measures").

© 2020 Wegwarth et al. *JAMA Network Open.*

eMethods 3. AAPOR Survey Disclosure Checklist

Results reported are from quantitative online survey research. No qualitative or content analyses were performed. The following information is disclosed in accordance with minimum disclosure requirements of the AAPOR Code for Professional Ethics and Practice.

**Basic disclosure items for quantitative survey research**	
Name of the survey sponsor	Max Planck Institute for Human Development, Berlin (Germany)
Name of the organization that conducted the survey	Online survey conducted by the market research institute company *infratest dimap*, Berlin(Germany)
Wording of the questions	Exact wordings of questions and stimuli provided in Part 2 of this **Supplement**
Population under study	German adults (≥ 18 years)
Sampling frame	Payback Panel of > 80,000 panelists recruited from members of the Payback Germany loyalty scheme (25 million German consumers)
Sample size	2,011
Mode of data collection	Online and by invitation
Type of sample (probability/non-probability)	Probability sample
Start and end dates of data collection	July 13–20, 2020
Location of data collection	Germany-wide
Response rate	71.8% (invited: 3,182; did not respond: 744; excluded due to failing quality check: 380; did not complete survey: 47; responses: 2,011; response rate: 2,011/[3,182 − 380])
Margin of sampling error	Percentage points at the 95% confidence level are reported for each outcome in the Results section
Are data weighted?	Yes, data were weighted by age, gender, region, household size, and education according to the 2018 Microcensus of the German Federal Statistical Office, which covered the German population eligible to vote
Contact for more information	PD Dr O Wegwarth<br>Max Planck Institute for Human Development (Berlin, Germany)<br>wegwarth@mpib-berlin.mpg.de

© 2020 Wegwarth et al. *JAMA Network Open.*
